# Genome-wide analysis of the soybean *eEF* gene family and its involvement in virus resistance

**DOI:** 10.3389/fpls.2024.1421221

**Published:** 2024-08-19

**Authors:** Hexiang Luan, Daiqiao Song, Kai Huang, Shuxin Li, Hao Xu, Pradeep Kachroo, Aardra Kachroo, Longgang Zhao

**Affiliations:** ^1^ Institute of Plant Genetic Engineering, College of Life Sciences, Qingdao Agricultural University, Qingdao, Shandong, China; ^2^ College of Food Science and Engineering, Qingdao Agricultural University, Qingdao, China; ^3^ Department of Plant Pathology, University of Kentucky, Lexington, KY, United States; ^4^ College of Grassland Science, Qingdao Agricultural University, Qingdao, China

**Keywords:** GmeEF, eEF1A, bioinformatics, soybean, virus infection

## Abstract

Eukaryotic elongation factors (eEFs) are protein factors that mediate the extension of peptide chain, among which eukaryotic elongation factor 1 alpha (eEF1A) is one of the most abundant protein synthesis factors. Previously we showed that the P3 protein of Soybean mosaic virus (SMV), one of the most destructive and successful viral pathogens of soybean, targets a component of the soybean translation elongation complex to facilitate its pathogenesis. Here, we conducted a systematic analyses of the soybean *eEF* (*GmeEF*) gene family in soybean and examinedits role in virus resistance. In this study, GmeEF family members were identified and characterized based on sequence analysis. The 42 members, which were unevenly distributed across the 15 chromosomes, were renamed according to their chromosomal locations. The GmeEF members were further divided into 12 subgroups based on conserved motif, gene structure, and phylogenetic analyses. Analysis of the promoter regions showed conspicuous presence of myelocytomatosis (MYC) and ethylene-responsive (ERE) cis-acting elements, which are typically involved in drought and phytohormone response, respectively, and thereby in plant stress response signaling. Transcriptome data showed that the expression of 15 *GmeEF* gene family members changed significantly in response to SMV infection. To further examine EF1A function in pathogen response, three different Arabidopsis mutants carrying T-DNA insertions in orthologous genes were analyzed for their response to Turnip crinkle virus (TCV) and Cucumber mosaic virus (CMV). Results showed that there was no difference in viral response between the mutants and the wild type plants. This study provides a systematic analysis of the *GmeEF* gene family through analysis of expression patterns and predicted protein features. Our results lay a foundation for understanding the role of *eEF* gene in soybean anti-viral response.

## Introduction

Eukaryotic elongation factors (eEFs) is a GTP-binding protein and play a central role in the protein biosynthesis, which elongate the nascent polypeptide chain by one amino acid at a time. In prokaryotic cells, there are three types of elongation factors, named EF-Tu, EF-Ts, and EF-G respectively and in eukaryotic cells, called eukaryotic elongation factor 1 alpha (eEF1A), eEF1B, and eEF2. eEF1A is a multimeric protein that with molecular masses of around 50 kDa and has three distinct domains: domain I (binds GTP), domain II (binds the aminoacyl end of the aminoacylated tRNA and actin), and domain III (binds actin) (Kanibolotsky et al., 2008; Soares et al., 2009; Abbas et al., 2015; Juette et al., 2022). Domain I is made up of Rossmann-fold topology. Domain II and domain III are made up of entirely from beta-strands, each domain contains two beta sheets that form a beta barrel (Soares et al., 2009). eEF1A is an important and highly conserved protein with a typical role in binding and delivering aminoacylated tRNA to the A-site of the ribosomes in a GTP-dependent manner. After tRNA transfer, eEF1A is recycled to its active GTP-bound form by the guanine nucleotide exchange factor, eEF1B (Gromadski et al., 2007; Maruyama et al., 2019; Mills and Gago, 2021). eEF1A proteins are multifunctional because they have been implicated in non-canonical roles besides its function protein synthesis. It has been showed that eEF1A participate in the growth and proliferation of cells, including construction of cytoskeleton, nucleocytoplasmic trafficking, endoplasmic reticulum (ER) stress, signal transduction, apoptosis, and protein degradation (Hetherington et al., 2016; Luan et al., 2016; Migliaccio et al., 2016; Małecki et al., 2017; Liu et al., 2020; Liu et al., 2019; Akram et al., 2020; Hou et al., 2021; Romaus-Sanjurjo et al., 2022).

Chaperone activity of elongation factors may be important in protecting the plants from stress factors of the environment that lead to denaturation of proteins. eEF1A may be involved in the response process of plants to stress, which is closely related to the plant resistance, such as high temperature, high salinity, and drought (Guo et al., 2014; Momcilovic et al., 2016; Djukić et al., 2019; Sun et al., 2020; Markovic et al., 2021). Identically, a body of data demonstrates the potential role of components of the eEF1A in plant virus infection. Their functions mainly include promoting viral RNA translation, regulating viral RNA replication, and accelerating viral infection. The work reveals that silencing of a homolog of the *eEF1A* (*NbS00023178g0001.1*) suppressed both Tomato spotted wilt virus (TSWV) disease symptom development and systemic spread of the virus (Garcia-Ruiz, 2019; Helderman et al., 2021). eEF1A can facilitate Chinese wheat mosaic virus (CWMV) infection in plants via its binding to the 3′-untranslated region (UTR) of CWMV genomic RNAs (Chen et al., 2021); eEF1A binds to the 3′-end of the Tomato bushy stunt virus (TBSV) RNA as well as to TBSV p33 replication co-factor to enhanced TBSV replication (Li et al., 2009; Gamarnik et al., 2010). There is verified enhanced susceptibility to Cucumber mosaic virus (CMV) and Tobacco rattle virus (TRV) in LreEF1A4-overexpressing transgenic petunia plants (Sun et al., 2020). Research shows that eEF1A can not only promote virus replication but also inhibit virus replication. The study demonstrated the key role of the *eEF1A* gene in sustaining the resistance against Tomato yellow leaf curl virus (TYLCV), most probably by inhibiting virus replication and/or movement (Wazwaz, 2013).

When reviewing the previous literatures, the controversy concerning the effect of eEF1As on virus propagation still exists, which eEF1A can both promote and inhibit virus infection. The mechanisms underlying these apparently conflicting findings are not yet well understood. What’s more, few studies have identified and characterized the *eEF1A* genes to clarify the mechanism of SMV infection. Here, we attempt to determine the role of the Arabidopsis eEF1A homologous mutant in the pathogenesis of SMV. Given the completion of soybean genome sequencing, we used soybean genome and transcriptome data to analyze the *eEF* family genes from the aspects of systematic evolution, gene structure, protein motif, and chromosome location and further analyzed the gene function of GmEF1A homologous mutants in Arabidopsis under CMV infection. This study provides a theoretical basis for further exploring the function of GmeEF1A resistance to virus.

## Materials and methods

### Plant growth conditions and pathogen infections

The T-DNA insertion mutants generated by the Salk Institute Genomic Analysis Laboratory were obtained from the Arabidopsis Biological Resource Center. The genotypes of three mutant lines: SALK_050704C (AT1G07930), SALK_079753C (AT1G07920), SALK_063369C (AT5G60390) were in the Col-0 ecotype background. Homozygous T-DNA insertion lines were generated by self-crossing heterozygous lines and verified by PCR amplification with primers specific for the T-DNA LP in combination with RP and RP in combination with LBB1 on inserted genes. Arabidopsis mutants that were grown in MTPS 144 Conviron (Winnipeg, MB, Canada) walk-in-chambers at 22°C, 65% relative humidity, and 14 h photoperiod. Soybean [*Glycine max* (L.)] cultivar NN1138-2 were grown in an aphid-free greenhouse at 25°C day and 20°C night, in 65% relative humidity and during a 14 h photoperiod. Transcripts synthesized *in vitro* from a cloned cDNA of TCV using T7 RNA polymerase were used for viral infections. For inoculations, 5 mg of the viral transcript was suspended in 80 μl inoculating buffer and DEPC water up to 200 μl. Healthy leaves of Arabidopsis were inoculated with 2μl inoculating mixture by rub-inoculation and then transferred to a Conviron MTR30 reach-in chamber.

### Identification and bioinformatics analysis of *eEF* family genes in soybean

First, we searched the genes in soybean transcriptome data to obtain gene ID and then searched in NCBI to obtain the eEF genes and protein sequences. Next, the physical and chemical properties of eEF proteins including the molecular weights (in kDa) and isoelectric points (pI), were analyzed using ExPASy (https://web.expasy.org/compute_pi/). Motif location was analyzed using MEME (https://meme-suite.org/meme/doc/meme.html?man_type=web). The conserved domains of eEF protein were evaluated using conservative structure domain tools in the NCBI database (https://www.ncbi.nlm.nih.gov/Structure/cdd/wrpsb.cgi). Two thousand base pairs upstream regions from the start codon site of *GmeEF* genes were sumbitted to Plant CARE (http://bioinformatics.psb.ugent.be/webtools/plantcare/html/) to predict cis-element of *GmeEF*. The results of protein phylogeny, gene structure, and protein motif analysis were displayed using TBtools.

### Multiple sequence alignment and phylogenetic tree

The eEF protein sequences of rice and Arabidopsis were extracted from Ensembl plant database (https://plants.ensembl.org/) for phylogenetic analysis. The phylogenetic trees of eEF proteins from 42 *Glycine max*, 29 *Arabidopsis thaliana*, and 30 *Oryza sativa* were constructed using the MEGA7.0.26 adjacency method with the following parameters: neighbor-joining (NJ) method, JTT+G model, and 1000 bootstrap replicates. The trees were visualized and modified using FigTree version v1.4.4 (https://github.com/rambaut/figtree/).

### Analysis of the expression patterns

The RNA-seq data obtained from the JGI database (https://phytozome-next.jgi.doe.gov/report/gene/Gmax_Wm82_a2_v1/Glyma.02G182800) were used to explore the spatiotemporal expression patterns of *GmeEF1A* gene in pods, root-hairs, leaves, roots, nodules, seeds, shoot apical meristem(sam), stems, and flowers. The heat map of expression of *GmeEF1A* genes was plotted using TBtools.

Mechanical inoculation of SMV SC3 virus on soybean fully expanded unifoliate leaves. Samples were collected at 0 d, 7 d, and 14 d after treatment and stored at -80°C. Three biological replicates were analyzed for each treatment and control.

### Functional verification of AteEF1a in Arabidopsis thaliana mutant

The three Arabidopsis mutants were ordered from Arabidopsis Biological Resource Center and the homozygous were confirmed by the primers in **Supplementary Table 1**. For TCV or CMV inoculations, transcripts synthesized *in vitro* from a cloned cDNA of TCV using T7 RNA polymerase were used for viral infections (Dempsey et al., 1993; Oh et al., 1995). Both the TCV and CMV transcript were suspended in an inoculation buffer then were used for rub-inoculation of leaves. Samples collected include leaves inoculated for 2 d and 3 d, and systemic leaves inoculated for 6 d and 9 d for western blot analysis. Respectively spot-treated with 5, 10, 15, and 20 mM paraquat (20 μl droplets each/leaf) for cell death assays. Phenotypic changes were observed after 1 day of inoculation. For paraquat treatments, paraquat was prepared in sterile water and leaves were spot inoculated with 10 mL of 5, 10, 15, 25, and 50 mM solutions. Lesion sizes were measured 48 h after paraquat application using vernier calipers. Results presented are representative of two or three separate treatments.

## Results

### Identification and analysis of *eEF* family in soybean

Detailed information of GmeEFs, including gene name, gene ID, predicted protein length, molecular weight, and theoretical isoelectric point distribution is listed in **Table 1**. The 42 *GmeEFs* genes ranged in size from 465 (*GmeEF39*) to 4068 bp (*GmeEF42*) and the corresponding predicted proteins ranged from 154 (*GmeEF39*) to 1355 (*GmeEF42*) amino acids. GmeEF39 showed the lowest molecular weight of 16929.51 Da, while GmeEF42 had the highest molecular weight of 148018.65 Da. Their isoelectric points (pI) varied from 5.02 (GmeEF34) to 9.22 (GmeEF39) with 27 members exhibiting pI values < 7, and 15 members with pI values > 7, suggesting that most of these genes encode acidic proteins. In addition, the basic protein was mainly concentrated in groups D1, D3, and E2.

**Table 1:** *GmeEF* gene and predicted protein sequence characteristics.

Serial No.	Gene name	Gene ID	CDS (bp)	Length (aa)	MW (Da)	pI
1	*GmeEF1*	Glyma.02G182800	2031	676	7,4929.76	6.11
2	*GmeEF2*	Glyma.03G150600	1365	454	4,9315.48	6.4
3	*GmeEF3*	Glyma.04G188700	1433	480	5,2207.69	6.21
4	*GmeEF4*	Glyma.04G206300	2253	750	8,3832.38	7.52
5	*GmeEF5*	Glyma.05G041900	1430	479	5,2507.23	6.33
6	*GmeEF6*	Glyma.05G055500	2253	750	8,6083.1	5.41
7	*GmeEF7*	Glyma.05G089000	1344	447	4,9356.09	9.14
8	*GmeEF8*	Glyma.05G114900	1344	447	4,9263.99	9.15
9	*GmeEF9*	Glyma.05G163800	2382	793	8,6453.39	6.44
10	*GmeEF10*	Glyma.05G243400	1518	505	5,6228.27	5.08
11	*GmeEF11*	Glyma.06G159400	2064	687	7,6684.33	6.57
12	*GmeEF12*	Glyma.06G176900	1430	479	5,2129.58	6.21
13	*GmeEF13*	Glyma.06G285200	2013	670	7,2569.2	6.36
14	*GmeEF14*	Glyma.08G051200	1515	504	5,6292.5	5.15
15	*GmeEF15*	Glyma.08G121100	2364	787	8,5998.66	6.03
16	*GmeEF16*	Glyma.08G170000	2532	843	9,4043.26	5.8
17	*GmeEF17*	Glyma.08G171500	1401	466	5,0939.47	8.91
18	*GmeEF18*	Glyma.08G174200	3033	1010	1,07906.7	8.69
19	*GmeEF19*	Glyma.09G258100	2028	675	7,4904.73	7.94
20	*GmeEF20*	Glyma.09G283700	3042	1013	1,11992.54	6.3
21	*GmeEF21*	Glyma.10G103000	2043	680	7,5188.02	6.25
22	*GmeEF22*	Glyma.10G212900	1347	448	4,9500.23	9.15
23	*GmeEF23*	Glyma.10G225800	4035	1344	1,47072.33	5.2
24	*GmeEF24*	Glyma.12G120500	2013	670	7,2610	6.2
25	*GmeEF25*	Glyma.13G165400	2961	986	1,09947.06	5.08
26	*GmeEF26*	Glyma.13G210600	1353	450	4,9148.64	6.85
27	*GmeEF27*	Glyma.15G102200	1242	413	4,5253.99	5.58
28	*GmeEF28*	Glyma.15G253000	3048	1015	1,08343.26	8.46
29	*GmeEF29*	Glyma.15G255900	1401	466	5,0930.46	8.91
30	*GmeEF30*	Glyma.15G256800	2148	715	8,0309.56	7.6
31	*GmeEF31*	Glyma.15G256900	2484	827	9,1894.14	6.25
32	*GmeEF32*	Glyma.15G257100	2532	843	9,4057.29	5.8
33	*GmeEF33*	Glyma.16G068000	1344	447	4,9406.14	9.14
34	*GmeEF34*	Glyma.17G105700	2967	988	1,10118.17	5.02
35	*GmeEF35*	Glyma.17G124500	1344	447	4,8740.97	9.16
36	*GmeEF36*	Glyma.17G137600	2364	787	8,6899.97	5.53
37	*GmeEF37*	Glyma.17G186600	1344	447	4,9396.07	9.14
38	*GmeEF38*	Glyma.19G052400	1344	447	4,9279.99	9.15
39	*GmeEF39*	Glyma.19G078900	465	154	1,6929.51	9.22
41	*GmeEF40*	Glyma.19G111600	3069	1022	1,12792.39	5.85
41	*GmeEF41*	Glyma.19G153200	1359	452	4,9109.37	6.4
42	*GmeEF42*	Glyma.20G166200	4068	1355	1,48018.65	5.2

### Phylogenetic and structural analysis of the GmeEF proteins

**Figure 1:**
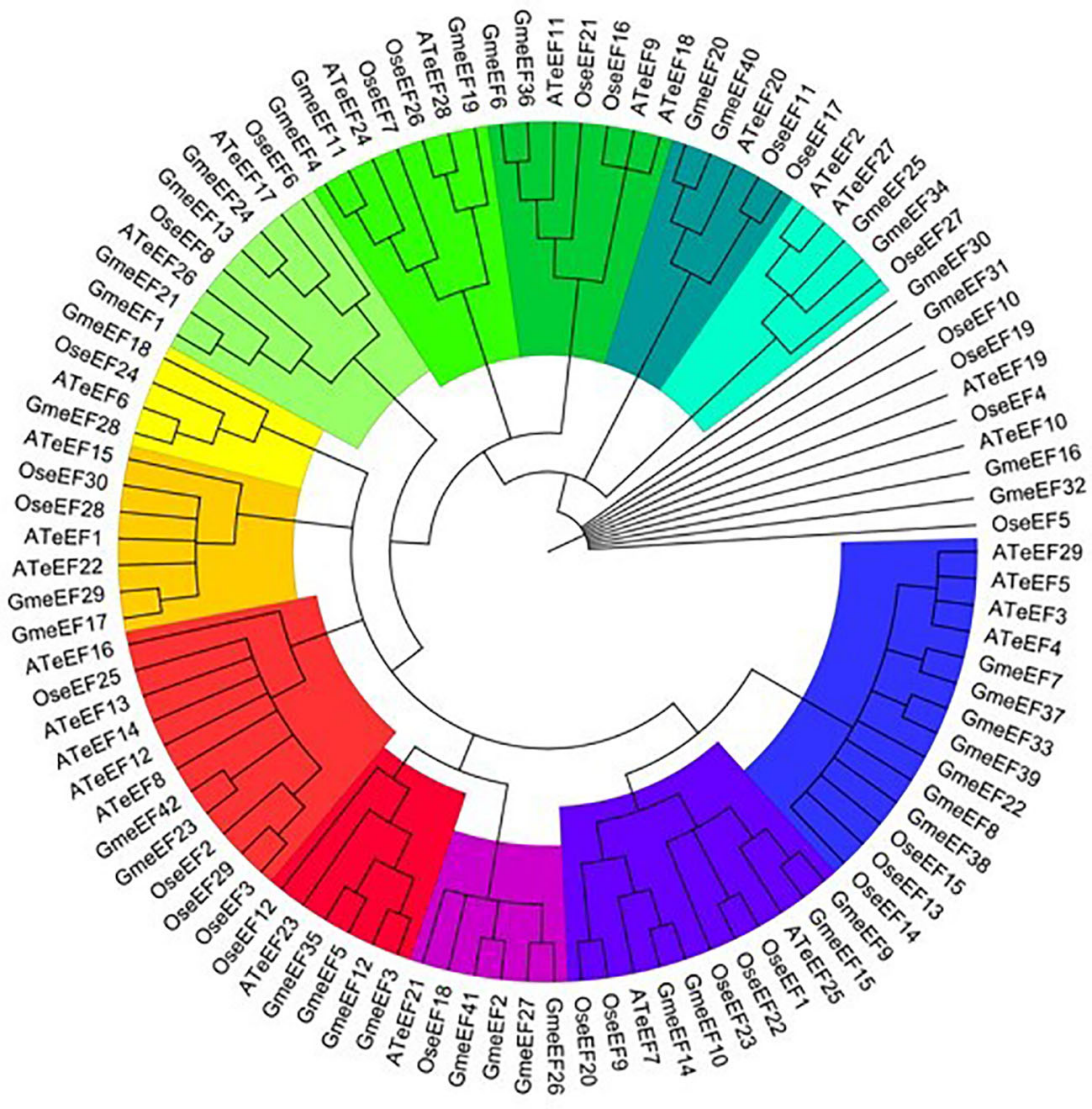
Phylogenetic tree of eEFs from *Glycine max*, *Oryza sativa*, and *Arabidopsis thaliana* was constructed by MEGA X according to NJ method. These proteins were divided into 12 subgroups and highlighted in different colors.

We next conducted phylogenetic analysis to investigate the evolutionary relationships amongst eEF members from *Glycine max*, *Oryza sativa*, and *Arabidopsis thaliana.* For this, we constructed a neighbor connection phylogenetic tree using MEGA X and default parameters. 29 genes from *Arabidopsis thaliana* and 30 genes from *Oryza sativa* were analyzed along with the 42 *GmeEF* genes (**Figure 1**).The results indicate that eEF from three species can be divided into four groups named A to E. Group D was the largest and was further divided into 5 subgroups. Subgroup E2 was the largest and was annotated as eEF1A by CD-Search. Group A, group B, and group E were divided into 2 subgroups, respectively. Group C was the smallest with only 8 eEFs members. Each subgroup had eEFs from all three species. Notably, GmeEF30, GmeEF31, GmeEF16, GmeEF32, OseEF10, OseEF19, OseEF4, OseEF5, AteEF10, and AteEF19 did not belong to any subtype of the gene family.

**Figure 2:**
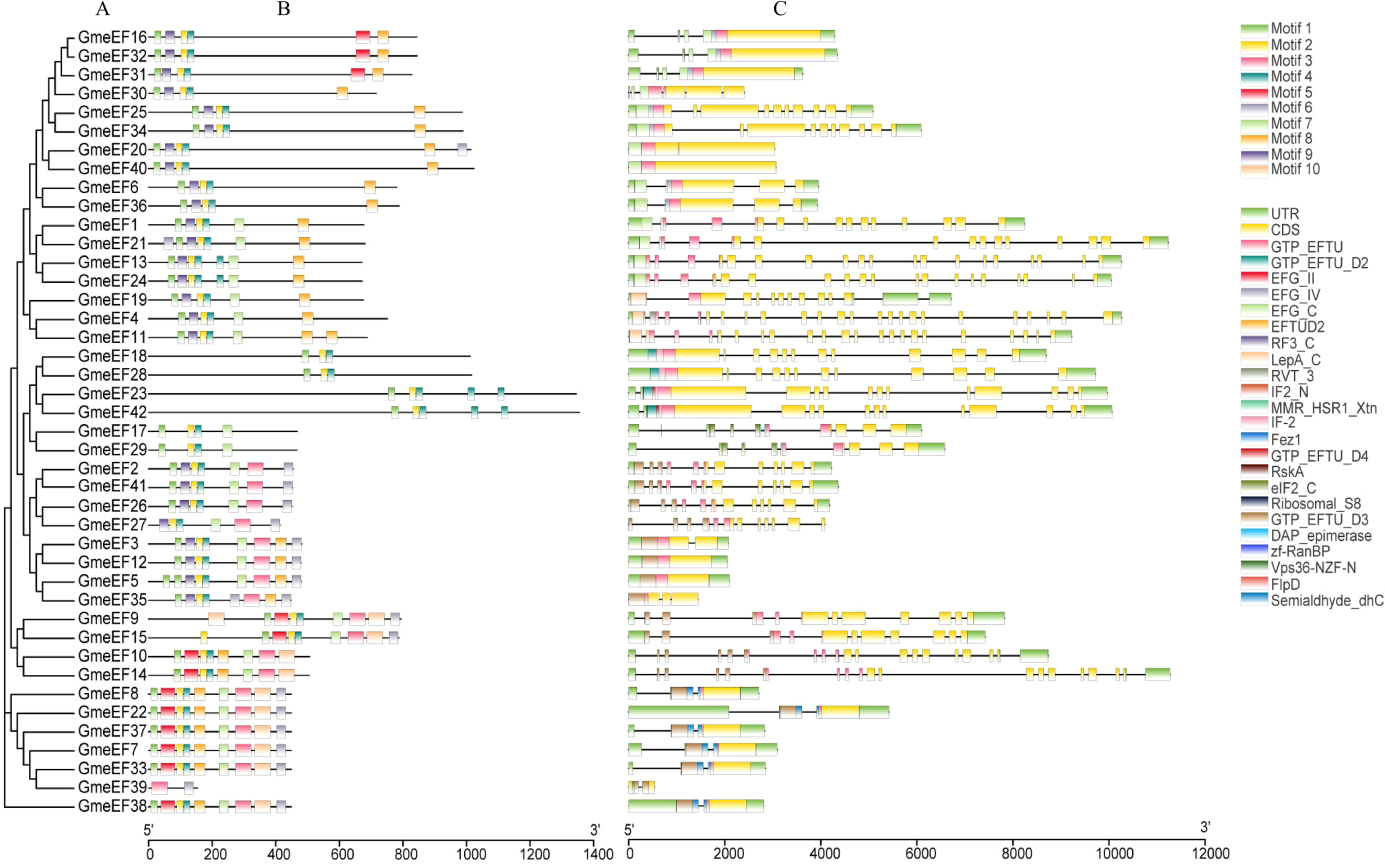
The phylogenetic tree, motif patten and gene structures of GmeEF. **(A)** The phylogenetic tree was constructed based on the full-length sequence of GmeEF proteins. **(B)** Conserved motifs of GmeEF proteins were identified by MEME suite. Each motif is represented by a specific color, with a total of 1–10 numbers. **(C)** Exon-intron organizations of *GmeEF* genes. Green boxes represent UTRs from 5’or 3’, yellow boxes represent CDS and black lines represent the introns.

The phylogenetic relationship of GmeEFs neighbor-joining phylogenetic tree was constructed by MEGA7.0 software with the Poisson model and 1000 bootstrap replications (**Figure 2A**). Multiple Expectation Maximization for Motif Elicitation (MEME) software identified 10 conserved motifs in the GmeEF family protein sequences (**Figure 2B**). Motifs 2 and 4 were found in all GmeEF sequences except GmeEF39, which only contained motifs 3 and 9. In addition, except GmeEF27 and 39, all family members contain motif 1. Presumably, motifs 1, 2, and 4 were relatively conserved during the expansion of the GmeEF family and played an important role in the functions of GmeEF proteins. Motif 10 was only found in members of group E. In group D, only the D4 subgroup contains motif 8. In addition, motif 8 was contained in all groups except group D, suggesting functional differences within GmeEF family members. Exon–intron structural diversity often plays a key role in the evolution of gene families and can provide additional evidence to support phylogenetic grouping. Structures of *GmeEF* genes clustered in the same clade were very close, including number and position of exons and introns (**Figure 2C**). The *GmeEF* gene family contained 0-22 introns, of which *GmeEF40*, *12*, and *5* were intronless. All genes in subgroup B2 contained the most introns, with the highest in *GmeEF4* (22 introns). It was speculated that most eEF members contain different transcripts.

### Chromosomal distribution analysis of *GmeEFs* genes

**Figure 3:**
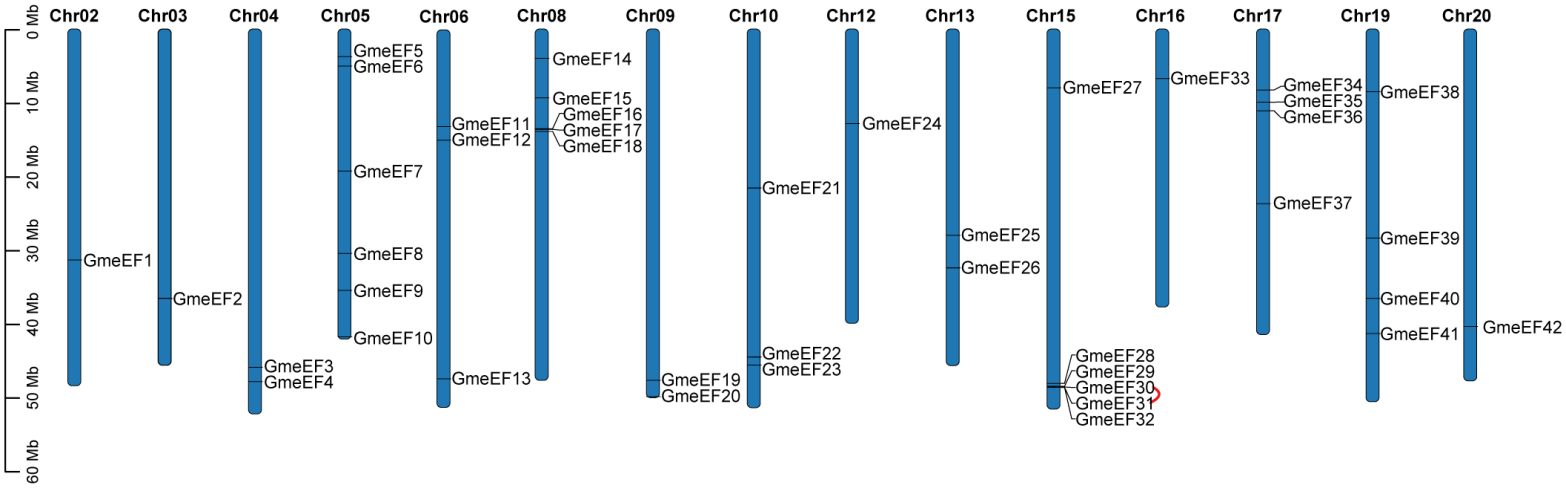
Mapping of *GmeEF* sequences on soybean chromosomes. The red sign represents a tandem duplication event region involving *GmeEF30* and Gme*EF31* on chromosome 15.

To identify members of the GmeEF family, we used local protein BLAST on GmeEFs protein sequences from soybean and conducted Hidden Markov Model (HMM) analyses with conserved model (SLAC1.hmm) as the query. A total of 42 predicted GmeEF proteins were identified and designated *GmeEF1-GmeEF42* based on their chromosomal locations (**Figure 3**). It was evident that the *GmeEFs* were unevenly distributed across the chromosomes. Specifically, chromosomes 5 and 15 contained the most *GmeEF* members with six; followed by chromosome 8 with five *GmeEF* members; chromosomes 17 and 19 with four *GmeEF* members each, chromosomes 6 and 10 each with three *GmeEF* members, chromosomes 4, 9, and 13 with two *GmeEF* members, chromosomes 2, 3, 12, 16, and 20 with one *GmeEF* gene each. No *GmeEF* genes were identified on chromosomes 1, 7, 11, 14, and 18. Moreover, one tandem duplication region comprising *GmeEF30* and *GmeEF31* was identified on chromosome 15.

### Analysis of promoter cis-acting elements of *GmeEF* genes in soybean

**Figure 4:**
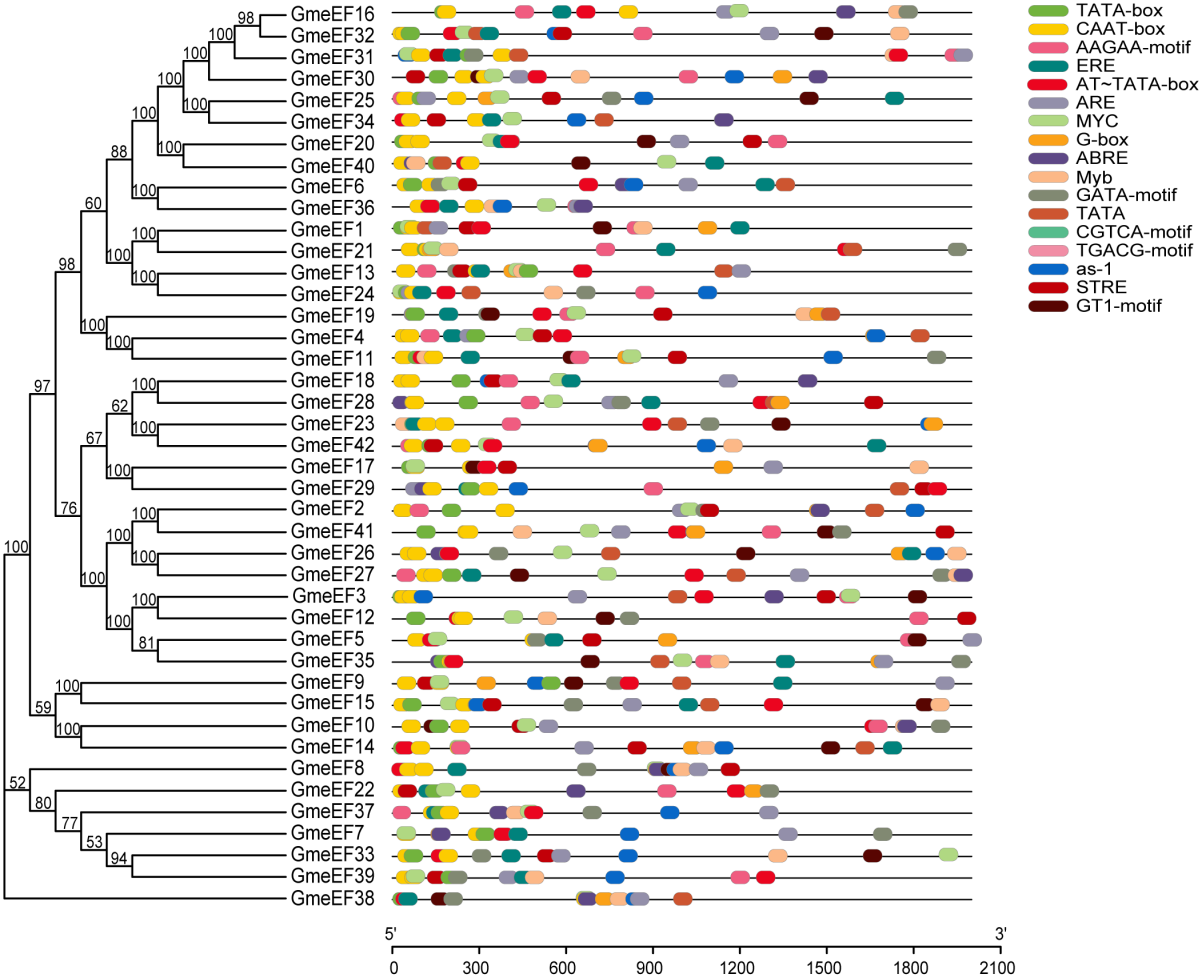
Distribution of cis-acting elements in soybean *GmeEF* promoter sequence. The figure shows 20 cis-acting elements that were selected and analyzed, and different colors indicate the elements related to growth and development, phytohormone responsive and stress responsive.

The control over gene transcription via upstream cis-acting regulatory elements (CAREs) is one of the most prominent mechanisms that regulates gene expression. To analyze the potential cis-elements, 2 kb regions upstream of *GmeEF* gene sequences were submitted to the Plant-CARE database. The type and position of cis-elements were marked as different icons. A total of 60 cis-acting elements were obtained, and 20 of them were selected for further analysis using TBtools (**Figure 4**). These cis-acting elements were related to phytohormone responsiveness, plant development, and stress responsive elements.

It is worth noting that the two promoters with the highest proportion in the GmeEF gene are CAAT box (45.32%) and TATA box (26.63%) (**Table 2**). Thus, CAAT and TATA box elements constituted the core promoter elements of *GmeEF* family members. Besides the cis-regulatory elements, AT~TATA-box, and TATA were also identified in majority of the *GmeEF* genes. Additionally, phytohormone responsive elements like methyl-jasmonate (MeJA)-responsive (CGTCA and TGACG), abscisic acid (ABA)-responsive element (ABRE), and ethylene-responsive elements (ERE) were identified. ERE participates in numerous development and stress responses (leaf development, senescence, fruit ripening, stimulation of germination, and oxidative stress). Other stress/development-associated motifs identified include, AAGAA-motif, GTI-motif, and activating sequence-1 (as-1); light response elements GATA-motif and G-box; drought-responsive myelocytomatosis (MYC) and myeloblastosis (MYB); antioxidant response element (ARE); and stress-responsive cis-elements (STRE). All in all, each *GmeEF* gene possessed different kinds and number of cis-elements, which are indicative of their unique functions or tissue-specific expression patterns.

**Table 2:** The detailed functional annotations of cis-elements.

Element name	Percentage	Function annotation
AAGAA-motif	1.08%	developmental-related elements
ABRE	1.84%	cis-acting element involved in the abscisic acid responsiveness
ARE	1.43%	cis-acting regulatory element essential for the anaerobic induction
as-1	0.79%	Stress response element
CAAT-box	26.63%	common cis-acting element in promoter and enhancer regions
CGTCA-motif	0.79%	cis-acting regulatory element involved in the MeJA-responsiveness
ERE	1.84%	ethylene-responsive element
G-Box	1.71%	cis-acting regulatory element involved in light responsiveness
GATA-motif	0.94%	part of a light responsive element
GT1-motif	0.84%	light responsive element
MYB	4.55%	abiotic stress responses
MYC	2.52%	abiotic stress responses
STRE	1.23%	Stress response element
TATA	0.68%	regulate transcription
TATA-box	45.32%	core promoter element around -30 of transcription start
TGACG-motif	0.79%	cis-acting regulatory element involved in the MeJA-responsiveness

### *GmeEF* gene expression pattern

**Figure 5:**
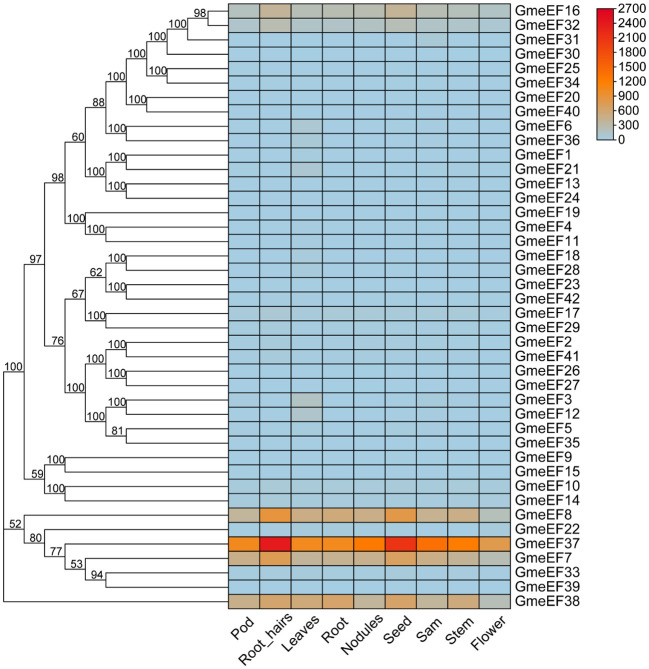
Expression analysis of *GmeEFs* during growth and development of different tissues of soybean. The RNA sequencing (RNA-Seq) dataset of 9 tissues of soybean collected from different development stages were obtained from JGI database to generate heatmap. The samples are listed at the bottom of each lane, and the color scale is shown at the right. The color box from blue to red indicate an increased expression level.

To further examine *GmeEF* gene function, we investigated their expression profiles in various organs and at different growth stages of soybean. The transcriptome data of *GmeEF* genes obtained from the JGI database was used for comprehensive transcriptome analysis of different tissues including pods, root-hairs, leaves, roots, nodules, seeds, shoot apical meristem(sam), stems, and flowers (**Figure 5**). According to the results, GmeEF8, GmeEF37, GmeEF7, and GmeEF38 showed relatively high expression in most tissues than others, which shared similar expression patterns in specific tissue and showed relatively high expression level in root-hairs and seeds. Among them, GmeEF37 had the highest expression. Interestingly, all four genes belong to the E2 subgroup, which shared similar expression patterns in specific tissue. The transcripts of the *GmeEF16*, *17*, and *32* genes were weakly expressed in all tissues. In addition to these, other family members also showed expression in leaf tissue, though their expression levels were negligible.

### The collinearity analysis of the chromosomes from *Glycine max*, *Oryza sativa*, and *Arabidopsis thaliana*

**Figure 6:**
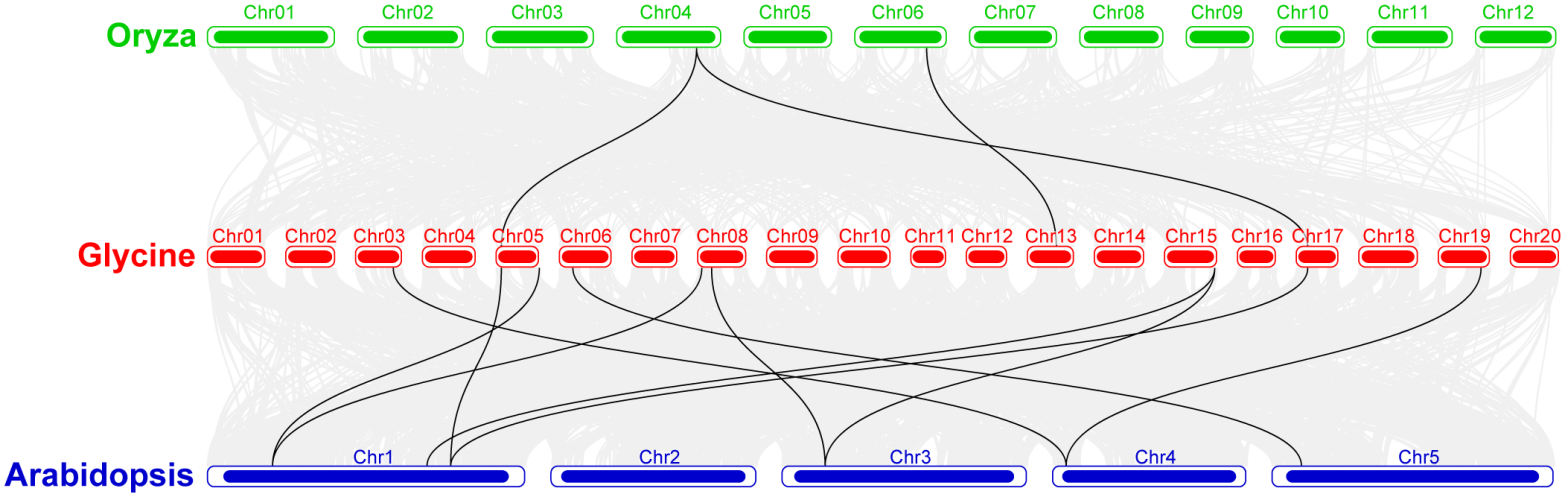
The collinearity analysis of the chromosomes from *Glycine max*, *Oryza sativa*, and *Arabidopsis thaliana.* The gray lines indicated all syntenic blocks within these three species genomes, and black lines indicated the syntenic *GmeEF* gene pairs.

Synteny analysis was conducted among the soybean, rice, and *Arabidopsis* sequences (**Figure 6**). The results showed that majority of the *Arabidopsis* genes showed synteny with *GmeEF* syntenic genes. In contrast, synteny was detected for only two *GmeEF* genes in rice. There were more collinear genes and blocks between soybean and *Arabidopsis* than between soybean and rice. We found 12 *GmeEFs* (*GmeEF1*, *GmeEF3*, *GmeEF4*, *GmeEF19*, *GmeEF20*, *GmeEF21*, *GmeEF22*, *GmeEF23*, *GmeEF24*, and *GmeEF42*) did not have orthologs in either rice or *Arabidopsis*. As a dicotyledonous plant, soybean possessed superior synteny with *Arabidopsis* than rice, indicating that most *eEF* genes were formed after the differentiation of dicotyledonous and monocotyledonous plants.

### Expression responses of *eEF1A* genes under virus infection in soybean and *Arabidopsis*

**Figure 7:**
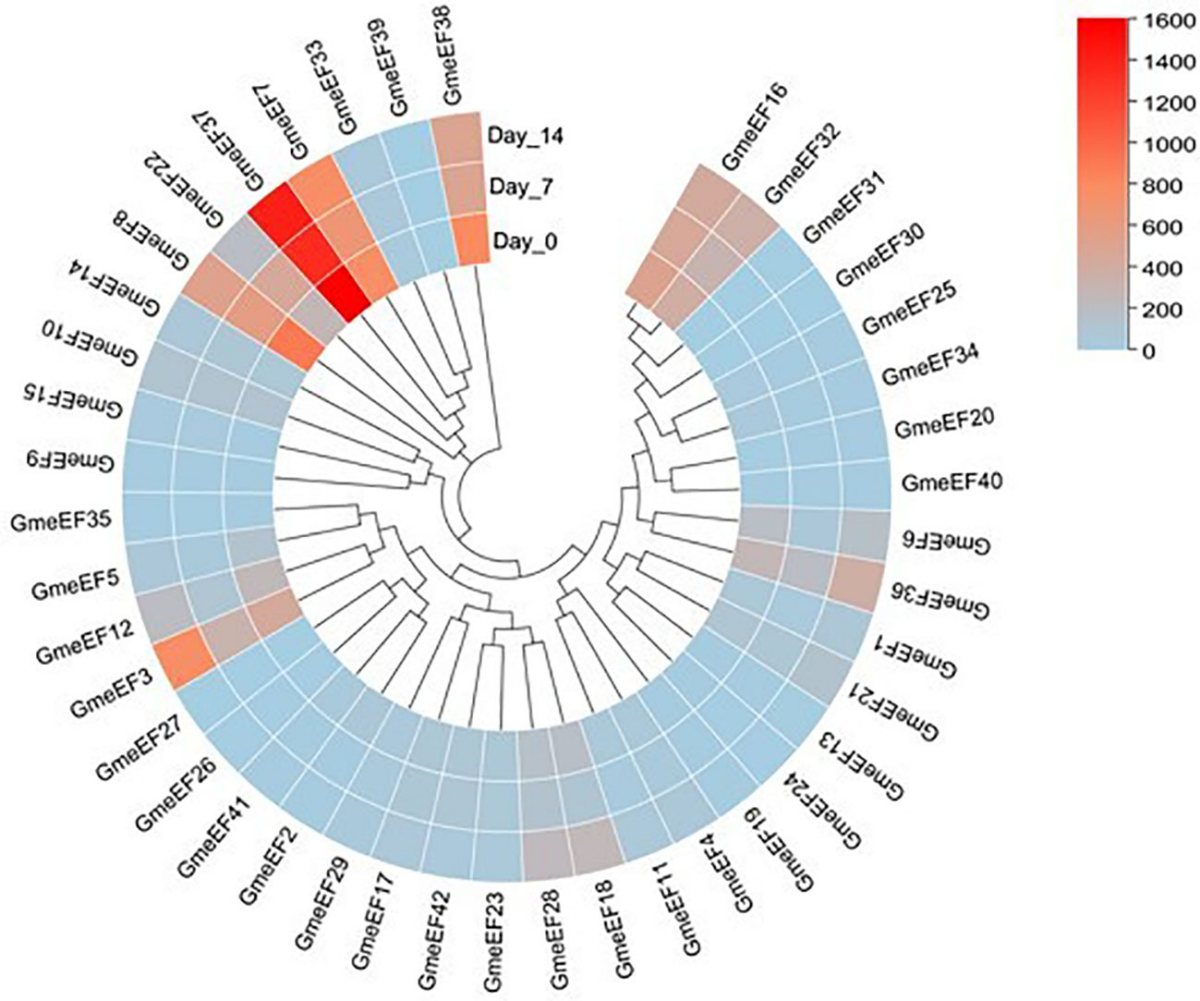
Expression analysis of *GmeEF* genes in response to the SMV strain SC3 infection. The heatmap of *GmeEF* genes was generated by the RNA-seq data which were obtained from the soybean plants inoculated with SMV SC3 at 0, 7, and 14 day post inoculation respectively. Three biological replicate experiments were performed for each treatment.

To investigate the involvement of *GmeEF* genes in SMV pathogenesis, we tested their expression at various time points post SMV infection (0, 7, and 14 dpi) using RNA-seq analysis. The results showed that a large number of genes were suppressed in response to virus infection. *GmeEF8* and *GmeEF38* were significantly downregulated at 7 dpi; *GmeEF22* presented unimodal change along with time, increasing on 7 dpi and then decreasing on 14 dpi; Eight genes (*GmeEF1*, *3*, *5*, *6*, *12*, *18*, *21*, *28*, *32*, and *36*) had an opposite trend of decreasing on 7 dpi and then increasing on 14 dpi. These genes may play different roles in the process of SC3 infection (**Figure 7**). There was no significant change in transcript levels of other genes, indicating that these genes may not involve in SC3 infection. Taken together, the variational expression of *GmeEF* genes under SMV treatment implied that this gene family involved in virus infection in a complicated way.

According to the summary of the systematic genome characterization of the eEF family, we could realize that the E2 subgroup members were mentioned many times, which harbored cis-acting elements related to stress resistance in most members. In addition, GmeEF8, GmeEF37, GmeEF7, and GmeEF38 had a relatively high expression in most tissues than others. In our previous research, the GmeEF37 transcript expression was induced after SMV infection and the knockout lines of GmeEF37 showed reduced amount of SMV, which indicated its negative regulation in SMV infection (Luan et al., 2016). Therefore, eEF1A was chosen for further studies in *Arabidopsis*.

**Figure 8:**
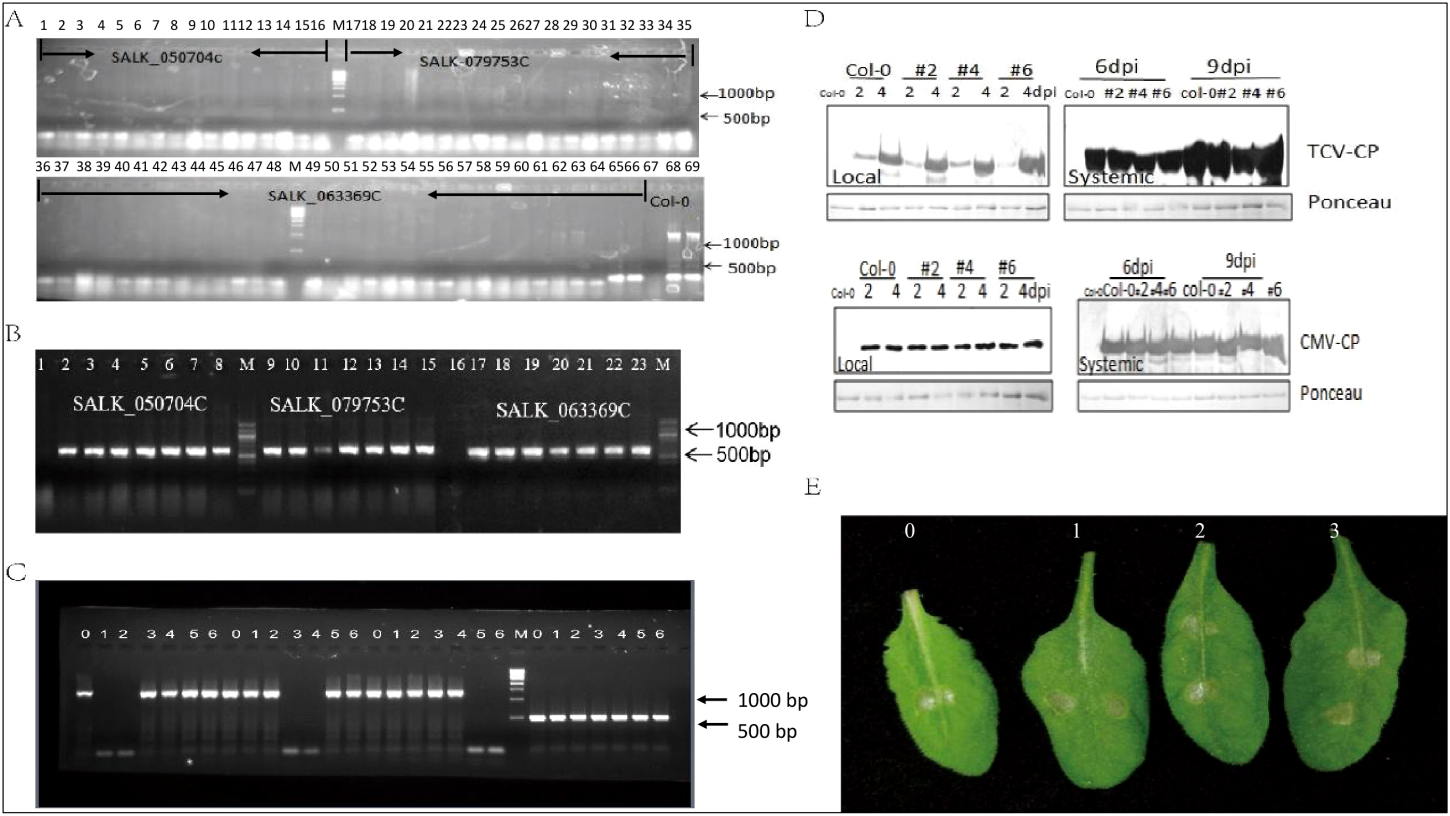
Elongation factor family function analysis. **(A)** Amplification of target genes by LP and RP primers: 1-16 SALK_050704C (AT1G07930); 16-35: SALK_079753C (AT1G07920); 36-66: SALK_063369C (AT5G60390). 67 empty; 68-69 Col-0. **(B)** Amplification of target genes expression by LBB1+RP. 1-8: SALK_050704C (AT1G07930); 9-15: SALK_079753C (AT1G07920); 17-23: SALK_063369C (AT5G60390). **(C)** Transcript level of 3 target genes. 0: Col-0; 1-2:SALK_050704C (AT1G07930); 3-4: SALK_079753C (AT1G07920); 5-6: SALK_063369C (AT5G60390). **(D)** Western blot analysis of TCV and CMV coat protein (CP) amount in three mutants. **(E)** Symptoms of three mutants after paraquat treatment, 0 is the Col-0, 1 is SALK_050704C, 2 is SALK_079753C, 3 is SALK_063369C.

Three homozygous SALK lines target to AteEF3 (AT1G07920), AteEF4 (AT1G07930), AteEF29 (AT5G60390) were genotyped by PCR with left border (LP) and right border (RP) primers as well as LBB1 and RP primers. Results showed that these three lines could not be amplified by LP and RP primers while the wide type plants showed band size over 1350 bp. The primer LBB1 in combination with RP could generate bands and wild type plants did not (**Figures 8A, B**). As we tested the expression of each specific genes in these three mutant lines, only the corresponding target gene was knocked out and the other two genes were not affected with the amplified size of 1350 bp length band, and tublin was control (**Figure 8C**). In order to explore the function of the Arabidopsis eEF1A in response to virus and chemical stress, the mutants were challenged with Turnip crinkle virus (TCV), Cucumber mosaic virus (CMV), and paraquat (1,1’-dimethyl-4,4’-bipyridinium dichloride) that induces cell death by promoting the formation of reactive oxygen species (Melicher et al., 2024). Western blot showed TCV and CMV accumulated the same amount in the local leaves of 2 and 4 dpi, as well as the distal leaves of 6 and 9 dpi of three mutant lines and did not show any difference with wild type plants (**Figure 8D**). The all mutants and wild type plants also exhibited impaired cell death when treated with paraquat (**Figure 8E**). All these data indicated that knocked out single copy of AteEF1A did not affect the replication of the TCV and CMV, as well as the response to paraquat. However complementation studies are required to make firm conclusions about functional similarities between soybean and Arabidopsis eEF1A proteins.

## Discussion

It was well recognized that the eEF1A family comprises multiple copies in cotton, *Arabidopsis*, tomato, lily, and cassava (Pokalsky et al., 1989; Xu et al., 2007; Ransom-Hodgkins, 2009; Suhandono et al., 2014; Sun et al., 2020). eEF1A was well-known as an important component for protein biosynthesis (Xu et al., 2022) and a host factor in viral pathogenesis. In this study, 42 eEF family members were identified and systematically analyzed in soybean, their gene characteristics, chromosomal locations, phylogenetic relationships, domain composition, and cis-elements were predicted, tissue expression patterns and response to virus infection were carried out, which provide an opportunity to better understand of the eEF proteins. In the present study, 42, 30, and 29 *eEF* genes were identified in soybean, rice, and *Arabidopsis* respectively. To explore the evolutionary process of *eEFs*, phylogenetic analysis was conducted with comparing the *eEF* genes of soybean, rice, and *Arabidopsis* revealing 5 groups designated A to D. Subsequently, 5 groups were divided into 12 subgroups. It is worth noting that GmeEF30, GmeEF31, GmeEF16, GmeEF32, OseeEF10, OseeEF19, OseeEF4, OseeEF5, AteEF19, and OseeEF10 do not belong to any subtype of the gene family. Based on this, we speculate that the common ancestor of eEFs may evolve independently among different species from monocotyledon to dicotyledon.

The domains and motifs of transcription factors play an important role in protein interaction and DNA binding (Corbella et al., 2021). GmeEFs were identified in soybean with ten motifs. Motifs 2 and 4 were found highly conserved in almost all of the GmeEFs. Gene structure analysis of *GmeEFs* revealed the intron-exon structure was highly conserved in the same group and had similar intron region distribution. Almost all *GmeEFs* contained one or more introns, except for *GmeEF40*, *12*, and *5* which were intronless, indicating GmeEFs might have functional diversity by multiple splicing during growth and development in plants. In addition, different kinds of cis-elements were analyzed including stress responsiveness, phytohormone response, light response, and growth regulation. We also collected transcriptome data from different growth stages and tissues of soybeans, i.e, pods, root hairs, leaves, roots, nodules, seeds, sam, stems, and flowers and conducted comprehensive transcriptome analysis. The results revealed that the expression patterns of *GmeEF* genes were diverse. *GmeEF16*, *GmeEF23*, *GmeEF8*, *GmeEF37*, *GmeEF7*, and *GmeEF38* had a relatively high expression in most tissues than others, which shared similar expression patterns in specific tissue and showed relatively high expression levels in root-hairs and seeds, suggesting that these subgroup members might play important roles in root-hairs and seed development. A small number of *GmeEF* genes were expressed in leaves and the amount of expression was also very small. The majority of the *GmeEF* gene family members showed little expression. The tissue-specific/preferential expressions of *eEF1A* genes were found in other plants. In *Arabidopsis thaliana*, the highest level of *eEF1A* expression was found in specific tissue types including seeds, embryos, and roots (Ransom-Hodgkins, 2009). It may be because there are more polyribosomes in the meristem or the region of rapid division.

It has been previously demonstrated that eEF1A is involved in the regulation of responses to abiotic and biotic stresses. eEFs potential influence and connection with heat tolerance of plants has been the subject of many studies. The study have shown that a significant, positive, linear correlation was found between the expression of eEF1A and small grains productivity under heat-stress conditions (Djukić et al., 2019).eEF1A was upregulated in peanut worms from the M and L tidal flats, which response to sedimentary environments that have lower porosity and greater organic matter or even a high abundance of pathogens (Li et al., 2023). Virus invasion is a kind of biological stress. eEFs predominantly eEF1A, act in partially characterized complexes sometimes involving additional eEFs so as to enhance or inhibit virus replication (Chen et al., 2021; Helderman et al., 2021; Gan et al., 2024). Although plant viruses encode a number of essential proteins their genome is exceptionally small and they must depend on host resources and factors for their genome replication and movement (Wu et al., 2024). As an abundant, multifunctional cellular protein, the cellular factor eEF1A plays an important role in the regulation of F-actin stress fiber formation required for Respiratory syncytial virus assembly and release (Snape et al., 2018); eEF1A, an essential protein in the translation machinery, interacted with two proteins of a fish rhabdovirus, Siniperca chuatsi rhabdovirus, and inhibited virus infection via two different mechanisms (Meng et al., 2023);eEF1A and other subunits of the eEF1 complex have been suggested to be essential for RNA virus replication (Zeenko et al., 2002; Yamaji et al., 2006, Yamaji et al., 2009; Komoda et al., 2014). Both eEF1A and eEF1B play essential roles in the multiplication of Potato virus X (PVX) in pepper (Hwang et al., 2015); A2 of the four eEF1A was increased expression from infection with Tobacco mosaic virus (TMV) and Phytophthora infections in *Arabidopsis thaliana* (Ransom-Hodgkins, 2009); In soybean, the results of a previous study showed that SMV-P3 targets host elongation factors resulting in UPR, which in turn facilitates SMV replication (Luan et al., 2016). In this study, we investigated whether SMV SC3 would affect the expression patterns of *GmeEF* family genes. RNA-seq results showed that a large members of the *GmeEF* gene family were induced or repressed at 7 or 14 dpi. *GmeEF3* induced significantly on 14 dpi, and *GmeEF8* and *38* were significantly down-regulated on 7 dpi. The transcription levels of other genes remain unchanged under SC3 infection. The results indicated that the pathogenesis of the virus came from the complex interaction between SMV SC3 and eEFs.

In addition, several *eEF* genes were selected for functional analysis experiments. In order to explore the function of the *Arabidopsis* eEF1A in response to the virus and chemical stress, the mutants (At1G07920, At1G07930, and At5G60390) were challenged with TCV, CMV, and paraquat. Unfortunately, the knockout of a single copy of *AteEF1A* did not affect the replication of TCV and CMV as well as the response to paraquat. The completion of the virus life cycle depends on the intricate interplay between virus and host. However, our knowledge of the identities and functions of such host factors remains largely unknown. The dynamics of virus-host interaction networks will help us to better understand plant resistance responses and develop resistant cultivars to viruses.

## Conclusion

In this study, 42 *eEF* genes were identified in soybean. Subsequently, we performed a systematic genome characterization of the *eEF* family genes. These *GmeEF* genes were located on 15 chromosomes and divided into 12 subgroups according to the phylogenetic tree. According to the analysis of conservative domains, gene structures, cis-acting elements, and the expression of GmeEFs in different parts of different periods, we found that GmeEFs belonging to the same subgroup had similar gene structures and motif compositions. We found that the GmeEF family was highly expressed in root-hairs and seeds. The function of elongation factor family was analyzed in *Arabidopsis*. The expression of each specific gene was tested in the three EF1a mutants and results showed that each mutant only knock out the corresponding gene and had no effect on other copies. Three mutant lines were used to test response to TCV, CMV, and paraquat respectively. We found that there was no difference between the wild type plants and the mutants in the viral expression or morphology. In a word, EF1a family does not involved in pathogens response in *Arabidopsis*, or it may involve in, but a single mutant is not enough. Other isoforms may confer it defect. This has laid the foundation for the pathogenesis of SMV and provided new ideas for the prevention and treatment of SMV.

## Data Availability

The original contributions presented in the study are included in the article/**Supplementary Material**, further inquiries can be directed to the corresponding authors.
