# Supplementary material for: Genome-wide analysis of the soybean eEF gene family and its involvement in virus resistance
Source: Front Plant Sci. 2024 Aug 19;15:1421221. doi: 10.3389/fpls.2024.1421221 (PMC11366645; doi:10.3389/fpls.2024.1421221)
Supplement: Supplementary file 1 [file Table1.docx]

**Table S1.** Primer sequences used for knock-out analysis.

| Name | Primers |
| --- | --- |
| SALK_079753C | LP: ACATCTCGCCTTATTTTTGGC  RP: TCAGATTGGTAACGGTTACGC |
| SALK_050704C | LP: CTCTTGACCAGATCAACGAGC  RP: AAGTGGTATGCGTTGCTTTTG |
| *SALK_063369C* | LP: GGTGGGTACTCGGAGAAAGTC  RP: GATTACTGGTACCTCCCAGGC |
| LBB1: | GCGTGGACCGCTTGCTGCAACT |
| *β-tubulin* | CGTGGATCACAGCAATACAGAGCC  CCTCCTGCACTTCCACTTCGTCTTC |
